# Supplementary material for: Crosstalk with keratinocytes causes GNAQ oncogene specificity in melanoma
Source: eLife. 2021 Dec 23;10:e71825. doi: 10.7554/eLife.71825 (PMC8747508; doi:10.7554/eLife.71825)
Supplement: Supplementary file 2. — (a) Top 20 most highly expressed genes in mouse wildtype (WT) interfollicular epidermis (IFE) melanocytes. (b) Identification of GNAQ hotspot mutations among human malignant melanomas potentially arising in the epidermis. (c) Identification of GNA11 hotspot mutations among human malignant melanomas potentially arising in the epidermis. (d) Information on statistical tests in Figures 1—6. [file elife-71825-supp2.doc]

| **Rank** | **Gene symbol** | **Gene name** | **Directly related to pigment production/ melanosome biology?** | **Reference** |
| --- | --- | --- | --- | --- |
| **1** | Pmel | Premelanosome protein | Yes | (Lee et al., 1996) |
| **2** | Dct | Dopachrome tautomerase | Yes | (Tsukamoto et al., 1992) |
| **3** | Mir5133 | MicroRNA 5133 |  |  |
| **4** | Tyrp1 | Tyrosinase-related protein 1 | Yes | (Kobayashi et al., 1994) |
| **5** | Ptgds | Prostaglandin D2 synthase (brain) |  |  |
| **6** | Mlana | Melan-A | Yes | (Du et al., 2003) |
| **7** | Eef1a1 | Eukaryotic translation elongation factor 1 alpha 1 |  |  |
| **8** | Vim | Vimentin |  |  |
| **9** | Cd63 | CD63 antigen | Yes | (van Niel et al., 2011) |
| **10** | Krt14 | Keratin 14 |  |  |
| **11** | Lgals1 | Lectin, galactose binding, soluble 1 |  |  |
| **12** | Fth1 | Ferritin heavy polypeptide 1 |  |  |
| **13** | Rplp1 | Ribosomal protein, large, P1 |  |  |
| **14** | Gstp1 | Glutathione S-transferase, pi 1 |  |  |
| **15** | Slc24a5 | Solute carrier family 24, member 5 | Yes | (Lamason et al., 2005) |
| **16** | Gpnmb | Glycoprotein (transmembrane) nmb | Yes | (Loftus et al., 2009) |
| **17** | Rps27rt | Ribosomal protein, S27, retrogene |  |  |
| **18** | Ftl1 | Ferritin light polypeptide 1 |  |  |
| **19** | Krt10 | Keratin 10 |  |  |
| **20** | Rpl8 | Ribosomal protein L8 |  |  |

**Table 2a. Top 20 most highly expressed genes in mouse WT IFE melanocytes.**

**References for Table 2a:**

Du, J., Miller, A. J., Widlund, H. R., Horstmann, M. A., Ramaswamy, S., and Fisher, D. E. (2003). MLANA/MART1 and SILV/PMEL17/GP100 are transcriptionally regulated by MITF in melanocytes and melanoma. Am J Pathol 163, 333-43.

Kobayashi, T., Urabe, K., Winder, A., Jimenez-Cervantes, C., Imokawa, G., Brewington, T., Solano, F., Garcia-Borron, J. C., and Hearing, V. J. (1994). Tyrosinase related protein 1 (TRP1) functions as a DHICA oxidase in melanin biosynthesis. EMBO J 13, 5818-25.

Lamason, R. L., Mohideen, M. A., Mest, J. R., Wong, A. C., Norton, H. L., Aros, M. C., Jurynec, M. J., Mao, X., Humphreville, V. R., Humbert, J. E., et al. (2005). SLC24A5, a putative cation exchanger, affects pigmentation in zebrafish and humans. Science 310, 1782-6.

Lee, Z. H., Hou, L., Moellmann, G., Kuklinska, E., Antol, K., Fraser, M., Halaban, R., and Kwon, B. S. (1996). Characterization and subcellular localization of human Pmel 17/silver, a 110-kDa (pre)melanosomal membrane protein associated with 5,6,-dihydroxyindole-2-carboxylic acid (DHICA) converting activity. J Invest Dermatol 106, 605-10.

Loftus, S. K., Antonellis, A., Matera, I., Renaud, G., Baxter, L. L., Reid, D., Wolfsberg, T. G., Chen, Y., Wang, C., Prasad, M. K., et al. (2009). Gpnmb is a melanoblast-expressed, MITF-dependent gene. Pigment Cell Melanoma Res 22, 99-110.

Tsukamoto, K., Jackson, I. J., Urabe, K., Montague, P. M., and Hearing, V. J. (1992). A second tyrosinase-related protein, TRP-2, is a melanogenic enzyme termed DOPAchrome tautomerase. EMBO J 11, 519-26.

Van Niel, G., Charrin, S., Simoes, S., Romao, M., Rochin, L., Saftig, P., Marks, M. S., Rubinstein, E., and Raposo, G. (2011). The tetraspanin CD63 regulates ESCRT-independent and -dependent endosomal sorting during melanogenesis. Dev Cell 21, 708-21.

| **Cosmic Sample ID** | **PMID** | ***GNAQ* mutation** | **Other key mutations** | **Scope of study** |
| --- | --- | --- | --- | --- |
| 1175905 | 19078957 | *Q209L* | N/A | *GNAQ* only |
| 2543893 | 26275246: "*Malignant melanoma arising in blue nevus"* | *Q209L* | N/A | N/A |
| 2724266 | 28481359 | *Q209H* (*frq* =0.04) | N/A | N/A |
| 2719694 | 28481359 | *Q209L* | *BAP1* | MSK-Impact screen |
| 2718695 | 28481359 | *Q209L* | *SF3B1^R625C^* | MSK-Impact screen |
| 1993392 | 21726664 | *Q209L* | None | 30 gene screen |
| 1993559 | 21726664 | *Q209P* | None | 30 gene screen |
| 2088283-2088289  From one patient | 24504448 | *Q209P* | *BRAF^V600E^* | NGS |
| 2380390 (RP-A690) | 26091043 | *Q209P* | *SF3B1^R625H^* | NGS (TCGA-SKCM) |
| 2121734 (ER-A2NF) | 26091043 | *Q209P* | *SF3B1^R625H^* | NGS (TCGA-SKCM) |
| 2380420 (BF-AAP8) | 26091043 | *Q209P* | *BAP1* | NGS (TCGA-SKCM) |

**Table 2b. Identification of *GNAQ* hotspot mutations among human malignant melanomas potentially arising in the epidermis.** The Cosmic database was searched for GNAQ Q209 or R183 missense mutations in tumors with the following criteria: Primary site: Skin and Histology: Malignant melanoma. Must have one of the following terms for Subhistology: Superficial spreading, Nodular, Spitzoid, Lentigo maligna, Acral lentiginous, Amelanotic, Epithelioid, Nevoid, or NS (Not Specified). (Excluded subhistology terms: Benign, Desmoplastic, Mucosal, Blue). Must have one of the following terms for Subsite: ear, lip, elbow, back, upper back, lower back, ankle, trunk, groin, hand, knee, chest, scalp, face, leg, shoulder, arm, breast, neck, flank, extremity, upper arm, forearm, foot, chronically sun exposed, intermittently sun exposed, eye, non chronically sun exposed or NS (Not specified). (Excluded subsite terms: mucosal, axilla, subungual, penis, nipple, vulva). The total number of *GNAQ* tested samples defined by these terms was 2753. Samples that could have arisen in the epidermis are highlighted in yellow. N/A, non-applicable; NGS, Next generation sequencing.

| **Cosmic Sample ID** | **PMID** | **GNA11 mutation** | **Other mutations** | **Scope of study** |
| --- | --- | --- | --- | --- |
| 2544691 | 26825879 | Q209L | None | 4 genes screened |
| 1838375 | 22817889:  "*uveal melanoma*" | Q209L | N/A | N/A |
| 2013581 | 22842228:  "*uveal melanoma*" | R183C | N/A | N/A |
| 2013602 | 22842228 | R183C | NRAS^Q61H^ | NGS exome |
| 2013703 | 22842228:  "*uveal melanoma*" | Q209L | N/A | N/A |
| 2013594 | 22842228 | Q209L | EIF1AX | NGS exome |
| 753596 | none | R183C | N/A - *Cell line* | N/A |
| 2237811 | 24714776:  *"most likely represents metastatic uveal melanoma"* | Q209L | N/A | N/A |
| 2380396 (RP-A6K9) | 26091043 | Q209L | BAP1 | NGS (TCGA-SKCM) |
| 2339744 (W3-A285) | 26091043 | R183C | NRAS^Q61R^ | NGS (TCGA-SKCM) |
| 2121738 (ER-A3ET) | 26091043 | Q209L | P53^Y220C^, PLCB4 splice | NGS (TCGA-SKCM) |
| 2121737 (ER-A3ES) | 26091043 | Q209L | SF3B1^R625H^ | NGS (TCGA-SKCM) |

**Table 2c. Identification of *GNA11* hotspot mutations among human malignant melanomas potentially arising in the epidermis.** The Cosmic database was searched for GNA11 Q209 or R183 missense mutations in tumors with the following criteria: Primary site: Skin and Histology: Malignant melanoma. Must have one of the following terms for Subhistology: Superficial spreading, Nodular, Spitzoid, Lentigo maligna, Acral lentiginous, Amelanotic, Epithelioid, Nevoid, or NS (Not Specified). (Excluded subhistology terms: Benign, Desmoplastic, Mucosal, Blue). Must have one of the following terms for Subsite: ear, lip, elbow, back, upper back, lower back, ankle, trunk, groin, hand, knee, chest, scalp, face, leg, shoulder, arm, breast, neck, flank, extremity, upper arm, forearm, foot, chronically sun exposed, intermittently sun exposed, eye, non chronically sun exposed or NS (Not specified). (Excluded subsite terms: mucosal, axilla, subungual, penis, nipple, vulva). The total number of *GNA11* tested samples defined by these terms was 2295. Samples that could have arisen in the epidermis are highlighted in yellow. N/A, non-applicable; NGS, Next generation sequencing.

**Table 2d. Details on statistical tests in Figures 1-6 (page 1/3).**

| **Figure** | **Comparison of:** | **Statistical test used:** | **Exact**  **p value:** | **Other values:** | **Degrees of freedom:** | **Definition of replicates:** |
| --- | --- | --- | --- | --- | --- | --- |
| **1B** | Average number of LacZ+ cells per scale in Wt vs GNAQ mice at 1 week of age | Unpaired t- test (two tailed) | 0.18 | t=1.491 | df=7 | Experiment performed on 4 Wt mice and 5 GNAQ mice.  At least 25 scales were examined per mouse (depending on the number of scales in the row). |
| **1B** | Average number of LacZ+ cells per scale in Wt vs GNAQ mice at 8 weeks of age | Unpaired t- test (two tailed) | 0.045 | t=2.367 | df=8 | Experiment performed on 5 Wt mice and 5 GNAQ mice.  At least 25 scales were examined per mouse (depending on the number of scales in the row). |
| **1D** | Percentage of scales affected by loss of boundaries at 8 weeks of age | Kolmogorov-Smirnov test | 0.18 |  |  | Experiment performed on 5 Wt mice and 5 GNAQ mice.  At least 25 scales were examined per mouse (depending on the number of scales in the row). |

| **1D** | Percentage of scales affected by loss of melanin at 8 weeks of age | Kolmogorov-Smirnov test | 0.048 |  |  | Experiment performed on 5 Wt mice and 5 GNAQ mice.  At least 25 scales were examined per mouse (depending on the number of scales in the row). |
| --- | --- | --- | --- | --- | --- | --- |
| **2B** | Percentage of tomato positive cells sorted from Wt vs GNAQ IFE | Unpaired t-test (two tailed) | 0.012 | t=3.132 | df=9 | Percentages measured in 6 Wt tails/FACS runs and 5 GNAQ tails/ FACS runs |
| **2C** | Survival of WT, GNAQ and BRAF IFE melanocytes plated on fibronectin | 2 way ANOVA with Tukey's multiple comparisons test | 3.3 x 10^-5^ for genotype  1.6 x 10^-5^  for time | Geisser-Greenhouse's epsilon = 0.3539 |  | Fraction of survival measured in 10 independent cell cultures derived from 3 or 4 mice of each genotype |

**Table 2d. Details on statistical tests in Figures 1-6 (page 2/3).**

| **2H** | Survival of WT vs GNAQ IFE melanocytes plated on MEFs | 2 way ANOVA with Tukey's multiple comparisons test | 0.48 for genotype  0.31  for time | Geisser-Greenhouse's epsilon = 0.3030 |  | Fraction of survival measured in 3 and 2 independent cell cultures derived from 3 GNAQ mice and 2 WT mice |
| --- | --- | --- | --- | --- | --- | --- |

| **3A** | Survival of WT melanocytes plated with IFE vs WT melanocytes plated on fibronectin | 2 way ANOVA | 0.0071  for culture condition  1.0 x 10^-5^  for time | Geisser-Greenhouse's epsilon = 0.3081 |  | Fraction of survival measured in 3 and 3 independent cell cultures derived from 6 Wt mice. |
| --- | --- | --- | --- | --- | --- | --- |
| **3B** | Survival of GNAQ melanocytes plated with IFE vs GNAQ melanocytes plated on fibronectin | 2 way ANOVA | 0.0053  for culture condition  0.00063  for time | Geisser-Greenhouse's epsilon =  0.3370 |  | Fraction of survival measured in 3 and 3 independent cell cultures derived from 6 GNAQ mice. |
| **3D** | Circularity of WT vs GNAQ melanocytes plated with IFE | Unpaired t-test (two tailed) | 0.000000050 | t=5.675 | df=193 | Averages calculated from 116 WT cells and 79 GNAQ cells from four independent culture experiments |
| **3E** | Protrusion length of WT vs GNAQ melanocytes plated with IFE | Unpaired t-test (two tailed) | 0.00041 | t=3.593 | df=207 | Averages calculated from 115 WT cells and 94 GNAQ cells from four independent culture experiments |
| **3F** | Number of protrusions in WT vs GNAQ melanocytes plated with IFE | Unpaired t-test (two tailed) | 0.0096 | t=2.615 | df=207 | Averages calculated from 115 WT cells and 94 GNAQ cells from four independent culture experiments |
| **3G** | Cell area of WT vs GNAQ melanocytes plated with IFE | Unpaired t-test (two tailed) | 2.6 x10^-13^ | t=7.862 | df=193 | Averages calculated from 116 WT cells and 79 GNAQ cells from four independent culture experiments |
| **3I** | Percentage of melanocytes cultured with IFE exhibiting fragmentation of dendrites | Unpaired t-test (two tailed) | 0.021 | t=2.875 | df=8 | Percentage calculated from 5 WT cultures and 5 GNAQ cultures (total cells tracked = 187 and 98) |

**Table 2d. Details on statistical tests in Figures 1-6 (page 3/3).**

| **4B** | Total distance traveled by WT vs GNAQ cells co-cultured with IFE | Unpaired t-test (two tailed) | 0.86 | t=0.1827 | df=114 | Averages calculated from57 WT cells and 59 GNAQ cells from four independent culture experiments |
| --- | --- | --- | --- | --- | --- | --- |
| **4C** | Directness of cell migration in WT vs GNAQ cells co-cultured with IFE | Unpaired t-test (two tailed) | 0.18 | t=1.359 | df=106 | Averages calculated from 59 WT cells and 49 GNAQ cells from four independent culture experiments |
| **4D** | Percentage of cells that divided during 20 hours observation, when co-cultured with IFE. | Ordinary 1 way ANOVA with Tukey's multiple comparisons test | 0.0040 overall |  |  | Averages calculated from 5 WT, 5 GNAQ and 3 BRAF independent cultures. Total cell number tracked: 187 WT cells, 98 GNAQ cells and 121 BRAF cells. |
| **4H** | Average time for cytokinesis in cells plated with IFE. | Ordinary 1 way ANOVA, with Tukey's multiple comparisons test | 3.0 x 10^-10^  overall |  |  | Average time calculated from 1 GNAQ, 11 WT and 14 BRAF cells that divided in 5, 5 or 3 independent culture experiments. |

| **5A** | Survival of WT melanocytes directly co-cultured with IFE vs transwell cultured with IFE | 2 way ANOVA | 0.022 for culture condition |  |  | Fraction of survival calculated from 3 and 3 independent cell cultures for each condition, derived from 6 WT mice. |
| --- | --- | --- | --- | --- | --- | --- |
| **5B** | Survival of GNAQ melanocytes directly co-cultured with IFE vs transwell cultured with IFE | 2 way ANOVA | 0.27 for culture condition |  |  | Fraction of survival calculated from 3 and 3 independent cell cultures for each condition, derived from 6 GNAQ mice. |
| **6C** | Cell fibrousness in WT vs GNAQ melanocytes plated with IFE | Unpaired t-test (two tailed) | 1.8 x 10^-12^ | t=7.544 | df=193 | Averages calculated from 116 WT cells and 79 GNAQ cells from four independent culture experiments |
